# Risk factors and clustering of mortality among older adults in the India Human Development Survey

**DOI:** 10.1038/s41598-022-10583-4

**Published:** 2022-04-22

**Authors:** Ronak Paul, Rashmi

**Affiliations:** 1 Department of Public Health and Mortality Studies, International Institute for Population Sciences, Mumbai, 400088 Maharashtra India; 2 Department of Population and Development, International Institute for Population Sciences, Mumbai, 400088 Maharashtra India

**Keywords:** Diseases, Health care, Risk factors

## Abstract

With wide socioeconomic mortality differential among older adults in India, a constant question of death clustering across high-risk families and communities arises. The present study uses a follow-up survey from India to investigate the socioeconomic, demographic and health predictors of old-age mortality clustering. Data of 16,964 older adults nested within 12,981 households from 2352 communities were used from India Human Development Survey (IHDS) round-I (2005) who were further tracked down in round-II (2012). Bivariate association between the determinants of old-age mortality was investigated using the log-rank test. The multivariate analysis involved estimating the random-intercept Weibull proportional hazard model with three levels—individual (level 1), family (level 2) and community (level 3). We analyzed the sensitivity of multivariate results to unobservable variable and selection biases using the e-value method. The empirical analysis confirms that the risk of mortality is significantly heterogeneous between the families. The health status of older adults and the family’s socioeconomic status in the early years emerged as prominent predictors of a longer lifespan. With a strong association between household income and mortality hazard risk, the present study urges early life interventions as those started in late-life might have negligible impact on keeping the older adults alive and healthy.

## Introduction

The famous phrase of Thomas Hobbes denotes human lives as “nasty, brutish and short”^1^. Historically, people used to survive merely 25 years from birth; however, with the evolution in living conditions of human society and a vast reduction in the mortality rates, average life expectancy at birth has grown nearly 45 years today^2^. Though initially, developed countries experienced such mortality reductions in a non-uniform manner, the mortality reduction in younger ages was comparably much higher than the others due to a decline in the fatality figure of infectious diseases^3^. Such progress was combined with the falling fertility rates and increasing life expectancy, leading to an increase in the share of the aged in the total population^4^.

As per the United Nations report, the aged population would increase from 0.7 billion (9% of the global population) in 2019 to 1.5 billion (16%) in 2050^5^. Developed countries that have already experienced the demographic transition try to break the stereotypes of the aged population being dependent, frail, and burdened for society. Policies are working towards promoting the wellbeing of older adults and promoting healthy aging among individuals^6^. However, such policy reforms are rare in developing nations where the focus is on fulfilling the development needs of children and youth. Notably, even if the pace of population aging started in the high-income countries, by 2050, 80% of the older population is expected to live in low-and-middle-income countries, which brings the old-age mortality issue to the central stage^6^.

A systematic review from developed and developing countries indicate that social determinants like socioeconomic, cultural and environmental conditions, living and working conditions, social and community networks, the lifestyle of individuals are prominent predictors of old-age mortality^7^. Two longitudinal studies from UK and USA found a long term impact (i.e. nearly 2 and 6 years respectively) of socioeconomic and health status of individuals with survival in old ages^8,9^. A follow-up study from Taiwan found that individuals with higher ages, poor schooling, consistent unemployment and poor life satisfaction in wave 1 have a higher probability of mortality in later periods^10^. However, the health status of individuals (in the form of activities of daily living conditions and self-reported chronic diseases) emerged as the primary predictor among all the other factors. Though marriage had a significant protective impact on the lives of individuals, males were less likely to be alive in follow-up surveys^10^. A study from Bangladesh provided evidence that being head of the household and residing with a spouse or son helped reduce mortality among older adults^11^. Studies from Ethiopia, New Zealand, Israel and the United States found that living in a rural area, having different ethnic groups and continents of origin, and experiencing financial hardship or stress can easily trigger mortality at older ages^12–15^. Such differences in mortality risks across socioeconomic, cultural and environmental conditions suggest unequal distribution of mortality risks among older adults. These deaths may be clustered among certain families and communities, putting them under the higher-risk categories.

In India, 60 years and above population is projected to rise 13.2% in 2031^16^. Lesser consideration towards the older adults can create a more significant loss in the future in the form of old-age mortality or repercussions like catastrophic health spending, social and financial insecurity and physical, social and emotional distress^17,18^. Few existing literatures from India have shown the effect of age, gender, caste and living standard on old-age mortality^19,20^. Studies have shown that prior co-morbidities among older adults have further worsened the old-age mortality risks. Thus, maintaining a healthy lifestyle that involves eating a balanced diet, physical activity, and avoiding substance abuse has contributed to fewer diseases, further reducing the mortality risk in older ages^6^. Despite knowledge of such determinants, the quality and quantity of life of older adults in India vary across families and communities. This brings the need to understand the risk factors for such unequal distribution of mortality risks among older adults by considering heterogeneity at the household and community levels. The present study improves upon the limitations of extant studies and aims to examine the risk factors of old-age mortality in India using a multilevel survival approach based on a nationally representative survey. Present study also uses the follow up survey data to indicate the predictors of old-age mortality and contributes to the recent literature in this area through robust evidence.

## Methods

### Data

This research article utilized the India Human Development Survey (IHDS) wave-I and wave-II, jointly administered by the National Council of Applied Economic Research (NCAER) and the University of Maryland. IHDS is a nationally-representative, multi-topic, large-scale survey that provides essential information on health and morbidity, education, employment and economic status, fertility and marital relations, and social capital of the Indian population. IHDS wave-I and wave-II were conducted during 2005 and 2012, respectively, across all India’s states and union territories except for Andaman & Nicobar Islands and Lakshadweep. Both waves of IHDS adopted a multistage stratified random sampling design, and further details on sampling design, data collection and informed consent are available elsewhere^21,22^. Notably, IHDS wave-II was a panel survey, which re-interviewed 83% of the original IHDS wave-I households. Further details regarding the IHDS wave-II panel component are available in the user guide^22^.

This study refers to persons aged 60 years and above as older adults. Intending to examine old-age mortality, this study utilized the tracking sheet data of IHDS wave-II from 2005 to 2012. Further, to explore the determinants of mortality in older adults, we merged the individual-, household- and community-level information from wave-I with the tracking sheet information in wave-II. The analytical sample of this study is 16,964 older adults residing in 12,891 families and nested within 2,352 communities in India.

### Mortality statement

The information regarding the mortality status of older adults was obtained from the IHDS wave-II tracking sheet data. With the aim of re-interviewing wave-I households during wave-II, the IHDS collected data on the status of all wave-I respondents during wave-II (this information comprised the tracking sheet data). Notably, during wave-II, IHDS gathered information of the survival status of respondents and the year of death prior to wave-II if respondents were not alive. Therefore, this information on survival status and survival time was used to analyze the mortality of older adults in India. All older adults who died during this period were coded as “Yes”; otherwise, they were coded as “No”.

### Statistical methods

At the outset, we examined the sample distribution of older adults. Next, we estimated the incidence rate of old-age mortality between 2005 and 2012 and grouped it by gender and age group. Further, we performed bivariate and multivariable analyses to achieve the study objectives. Note that the mortality data described in “Mortality statement” contain censored observations (those older adults who did not experience mortality between wave-I and wave-II and older adults who were lost to follow-up). Therefore, in the bivariate analysis, we calculated the mean survival duration of older adults across the categories of risk factors by accounting for censoring in the data^23^. Further, log-rank tests were performed to examine the association between the risk factors and older adults’ mortality status by adjusting for censored cases. Statistical details of the log-rank test are available elsewhere^23^.

The multivariable analysis involved estimating random-intercept parametric survival regression models. Survival regression models help utilize the information from censored records in the retrospective life-course data, thereby curtailing the loss of crucial information^23^. Notably, parametric survival regression models have the advantage of more efficiently utilizing the information from censored cases compared to semi-parametric regression models^23^. In the survival models, our event of interest is the binary survival status of the older adults between IHDS 2005 and 2012.

Additionally, parametric survival regression models allow us to choose the underlying statistical distribution of time-to-old-age mortality^23^. Based on theoretical knowledge and statistical evidence, we use the Weibull proportional hazard model in our study. The Weibull regression model is appropriate when the hazard of the failure event (here, risk of mortality) is either monotonically increasing or decreasing^23^. Based on existing knowledge of human mortality, we know that the risk of mortality rises steadily among older adults with progressing age^24,25^. A similar trend is observed in our data (see Fig. 2) of Indian older adults. Therefore, using the Weibull regression hazard model to estimate mortality risk among Indian older adults is theoretically justified^25^. The statistical fit of the models was examined by comparing the Akaike information criterion (AIC) and Bayesian information criterion (BIC) scores of the five prominent random-intercept survival regression models (Exponential, Weibull, Lognormal, Loglogistic and Gamma). We aim to use the model with the lowest AIC and BIC scores, as that would best fit the data.

In the random-intercept Weibull hazard model, we included individual (level 1), family (level 2) and community (level 3) as the three levels. 16,964 older adults from 12,981 families were nested within 2352 communities, forming a hierarchical structure in our study sample. In India, older adults from the same families of the same communities are likely to share the same socioeconomic characteristics and household environment, which means the mortality risk might also be shared. Estimating mortality hazard using standard survival regression would overestimate the risk in this scenario, and using a multilevel framework becomes necessary^26,27^. The statistical description of the three-level random-intercept survival regression model is given below:

$$h\left({t}_{ijk}\right)= {h}_{0}\left({t}_{ijk}\right) {e}^{({\beta }_{1}{x}_{1ijk}+{\beta }_{2}{x}_{2jk}+{\beta }_{3}{x}_{3k}+{s}_{k}+{c}_{jk}+{e}_{ijk})}$$

Here, ${s}_{k}$ is the level 3 residual (group effect at community-level), ${c}_{jk}$ is the level 2 residual (group effect at family-level) and ${e}_{ijk}$ is the level 1 residual (individual level). $h\left({t}_{ijk}\right) \mathrm{and} {h}_{0}\left({t}_{ijk}\right)$ are overall and baseline hazard of old-age mortality for ith persons belonging to the jth family of kth community. ${\beta }_{1}$,${\beta }_{2}$ and ${\beta }_{3}$ gives the hazard coefficient of old-age mortality for the person-level, family-level and community-level independent variables, respectively, given the effect of all other independent variables and the group-level effects remains constant.

The random-intercept regression models provide the Intraclass Correlation Coefficient (ICC) and Median Hazard Ratio (MHR), which measures the mortality clustering of older adults within the families and the communities, respectively. The family-level ICC measures the correlation in mortality risk among older adults belonging to the same family of the same community^27,28^. It is calculated as^29^:

$${ICC}_{fam}= \frac{{\sigma }_{f}^{2}+{\sigma }_{c}^{2}}{{\sigma }_{i}^{2}+{\sigma }_{f}^{2}+{\sigma }_{c}^{2}}$$

where, ${\sigma }_{i}^{2}$, ${\sigma }_{f}^{2}$, and ${\sigma }_{c}^{2}$ are the individual-, family- and community-level random-effect variance.

Equivalently, the community-level ICC denotes the correlation in mortality risk among older adults of the same community^27,29^. It is calculated as:

$${ICC}_{comm}= \frac{{\sigma }_{c}^{2}}{{\sigma }_{i}^{2}+{\sigma }_{f}^{2}+{\sigma }_{c}^{2}}$$

where the notations have the usual meaning. The ICC value lies between 0 and 1. The higher the value of ICC, the greater is the extent of mortality clustering at the respective levels.

Equivalently, the family-level (or community-level) MHR gives the median relative change in the hazard of the old-age mortality among all possible identical older adults pairs from two separate randomly selected families (or communities) that are ordered by mortality risk^30^. The family-level and community MHR is calculated as:

$${MHR}_{fam}= {e}^{0.95 * {\sigma }_{f}}$$

$${MHR}_{comm}= {e}^{0.95 * {\sigma }_{c}}$$

where the notations have the usual meaning. The value of MHR is always greater than or equal to one such that the higher the value, the more is the heterogeneity in the old-age mortality risk across clusters. Further statistical details regarding the ICC and MHR are available from the cited references.

Further, the multivariable association between the independent variables and old-age mortality risk was shown using hazard ratios (HR). The HR gives the hazard of old-age mortality compared to the baseline mortality risk among older adults belonging to a particular category of an explanatory variable when the effect of other explanatory variables and the community- and family-level variability remain constant^23^.

Moreover, sensitivity analysis was performed by inspecting the presence of unobservable variable bias in the adjusted hazard ratios using the e-value method^31,32^. The e-value method gives the e-value statistic, which is defined as the minimum strength of association (on the hazard ratio scale) that an unmeasured confounder would need to have with both the treatment and the outcome variables after adjusting for the effect of other independent variables, such that the treatment-outcome variable association is nullified^31^. Therefore, the higher the e-value, the more robust is the corresponding hazard ratio to unobserved variable bias. The statistical significance of the e-value was determined from the CI limit (nearest limit to the null value of 1.00)^32^. The CI limit was 1.00 if the e-value was not statistically significant at the 5% level^32^.

We checked and found that none of the multivariable models violated the multicollinearity assumption^33^. Unfortunately, IHDS does not provide sample weight in the tracking sheet data, and the study results are unweighted. Statistical significance was determined at the 5% level unless mentioned otherwise. Statistical estimations were performed using the STATA 14 software^34^.

### Explanatory variables

Existing studies have shown several factors which explain the mortality among older adults^7,9,19,20^. We included these variables, conditional to their availability in the IHDS dataset. All the below-mentioned characteristics were measured for the older adults during wave-I. The individual-level variables related to the older adults include:

- Age-group (in years) (60–69, 70–79, 80 and above).
- Gender (female, male).
- Cardiovascular diseases (no, yes).
- Hypertension (no, yes).
- Diabetes (no, yes).
- Respiratory illnesses (no, yes).
- Other chronic illnesses (no, yes).
- Activities of daily living (no disability, has disability).
- Smokes tobacco (no, yes).
- Drinks alcohol (no, yes).
- Marital status (currently married, widowed, currently not married).
- Level of education (more than 10 years of schooling, 6–10 years of schooling, less than 5 years of schooling, No formal schooling).
- Working status (working, not working).
- Participates in social groups (yes, no).
- Headship status (household head, not household head).

The household-level variables considered in our study are:

- Family structure (single generation, nuclear, joint/extended). It was prepared from the information on household members and their relationship with the household head.
- Number of children in the household (three and more, two, one, none).
- Household wealth quintile (richest, rich, middle, poor, poorest). The household wealth quintile for wave-I was calculated using principal component analysis using the available information on household asset ownership. We used standard procedures documented elsewhere^35^.
- Household poverty (not below poverty line, below poverty line).
- Caste of household head (others, other backward classes, scheduled castes, scheduled tribes). The caste system is a form of social hierarchy exclusive to India. Constitutionally, three distinct social groups are recognized in India—scheduled tribes (ST), scheduled castes (SC) and other backward classes (OBC). The ST (predominantly tribal) and SC categories comprise the most socially backward. They traditionally belonged to the lowest rung of India’s now-defunct caste system. People of the OBC category, as the name implies, are members of a socially and economically backward community. However, their circumstances are better than those of the SC/ST population. The “Others” category consists of all people who do not belong to the three caste groups.
- Religion of household head (Hinduism, Islam, Others).

Taking a cue from extant research, we included three community contextual variables^36–38^:

- Education level of community (low, medium, high).
- Poverty status of community (low, medium, high).
- Social standard of community (low, medium, high).

We constructed these three community contextual characteristics by aggregating the information on the education level of individuals, BPL status of household and caste of the household to the community level, respectively. Prior to aggregation, we constructed binary variables of each of the three characteristics. Community education level was defined as the proportion of individuals with more than 10 years of schooling among all individuals in the community. The higher the proportion of educated individuals, the greater the community's education standard. The community poverty status was defined as the proportion of BPL households among all households in the community. A higher proportion of below poverty line households means a greater prevalence of poverty in the community. Further, community social standard was constructed as the proportion of Non-SC/ST households among all households in the community. Therefore, the higher the proportion of Non-SC/ST households, the greater is the community’s social standard. For ease of interpretation, we categorized the proportions into three categories—“low” (lowest 33rd percentile), “medium” (middle 33rd percentile), and “high” (highest 33rd percentile).

Additionally, we included the following community-level characteristics:

(d) Type of community (urban, rural).
(e) Geographical region (southern, western, eastern, central, north eastern, northern). The geographical regions divided India’s erstwhile 33 states and union territories into six areas based on administrative divisions^39^.

### Ethics approval and consent to participate

The present study utilized a publicly available secondary dataset with no information that would lead to the identification of the respondents. IHDS obtained the informed consent of respondents before the data collection. Therefore, no ethical approval was necessary for using these datasets. All survey methods were performed following the relevant guidelines and regulations.

## Results

### Sample description

Table 1 shows the characteristics of 16,964 older adults aged 60 years and above during IHDS 2005. Nearly 61% of older adults were aged between 60 and 69 years, and 50% were male. Nearly 6% and 4% of older adults had hypertension and diabetes, respectively. Moreover, one in ten older adults faced difficulty performing activities of daily living, one-fifth of older adults smoked tobacco, and 7% consumed alcohol. Further, six in ten older adults had no formal schooling, and 36% were widowed. While one-tenth of older adults lived in single generation households, 32% belonged to the lowest 40% wealth quintile households. Coming to the community context, we observed that 70% of older adults resided in rural areas, three in ten older adults belonged to communities with a high level of education and social standard. Further, 35% and 33% of children were from communities with low socioeconomic status and had a low maternal education level, respectively. In terms of population distribution, most older adults (33%) were from the Northern region, followed by the Southern (24%) region.

**Table 1:** Absolute (N) and percentage (%) distribution of older adults in India by individual-level, household-level and community-level characteristics during 2005.

Characteristics	Older adults (60 + years) in round-I	
	N	Col_%
***Individual-level characteristics***		
**Age-group (in years)**		
60–69	10,343	61.0
70–79	4912	29.0
80 and above	1709	10.1
**Gender**		
Female	8504	50.1
Male	8460	49.9
**Cardiovascular diseases**		
No	16,617	98.0
Yes	347	2.0
**Hypertension**		
No	15,862	93.5
Yes	1102	6.5
**Diabetes**		
No	16,346	96.4
Yes	618	3.6
**Respiratory illnesses**		
No	16,343	96.3
Yes	621	3.7
**Other chronic illnesses**		
No	15,974	94.2
Yes	990	5.8
**Activities of daily living**		
No disability	15,056	88.8
Has disability	1908	11.2
**Smokes tobacco**		
No	13,827	81.5
Yes	3137	18.5
**Drinks alcohol**		
No	15,755	92.9
Yes	1209	7.1
**Marital status**		
Currently married	10,645	62.8
Widowed	6102	36.0
Currently not married	217	1.3
**Level of education**		
More than 10 years of schooling	1274	7.5
6–10 years of schooling	2533	14.9
Less than 5 years of schooling	2939	17.3
No formal schooling	10,218	60.2
**Working status**		
Working	6779	40.0
Not working	10,185	60.0
**Participates in social groups**		
Yes	5981	35.3
No	10,983	64.7
**Headship status**		
Household head	8103	47.8
Not household head	8861	52.2
***Family-level characteristics***		
**Family structure**		
Single generation	1834	10.8
Nuclear	1870	11.0
Joint/extended	13,260	78.2
**Number of children in household**		
Three and more	4553	26.8
Two	3412	20.1
One	2979	17.6
None	6020	35.5
**Household wealth quintile**		
Richest	4578	27.0
Rich	3644	21.5
Middle	3323	19.6
Poor	2818	16.6
Poorest	2601	15.3
**Household poverty**		
Not below poverty line	13,507	79.6
Below poverty line	3457	20.4
**Caste of household head**		
Others	6079	35.8
Other backward classes	6792	40.0
Scheduled castes	3030	17.9
Scheduled tribes	1063	6.3
**Religion of household head**		
Hinduism	13,899	81.9
Islam	1648	9.7
Others	1417	8.4
***Community-level characteristics***		
**Education level of community**		
Low	5506	32.5
Medium	6354	37.5
High	5104	30.1
**Poverty status of community**		
Low	5380	31.7
Medium	5977	35.2
High	5607	33.1
**Social standard of community**		
High	5671	33.4
Medium	5874	34.6
Low	5419	31.9
**Type of community**		
Urban	4841	28.5
Rural	12,123	71.5
**Geographical region**		
Southern	4038	23.8
Western	2495	14.7
Eastern	2528	14.9
Central	1728	10.2
North Eastern	509	3.0
Northern	5666	33.4
**Overall**	**16,964**	**100.0**

N, sample; Col_%, column percentage.

**Figure 1:**
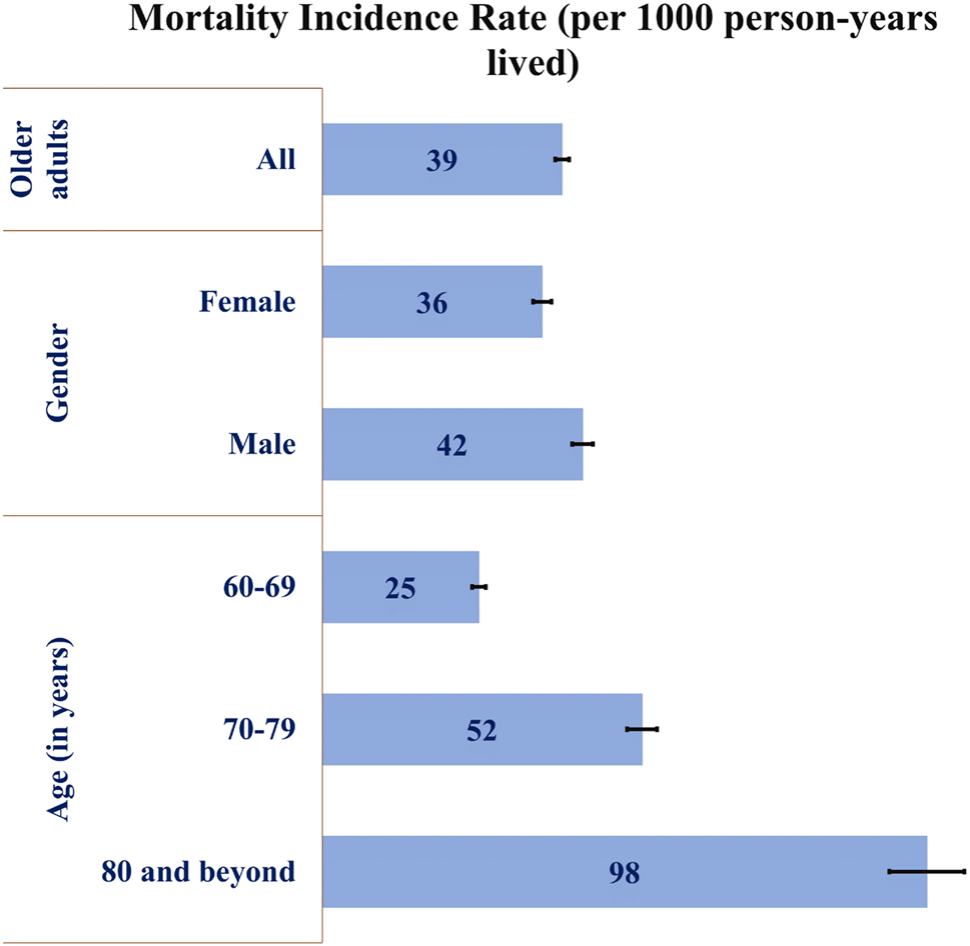
Incidence rate of mortality (deaths per 1000 person years lived) among older adults for the period from 2005 to 2012.

Figure 1 shows the Mortality Incidence Rate (per 1000 person-years lived (PYL)) among subgroups of older adults for 2005–2012. The overall old-age mortality rate was 39 per 1000 PYL. The mortality rate was higher in male older adults (42 deaths per 1000 PYL) and those aged 80 years and beyond (98 deaths per 1000 PYL) compared to their counterparts from other sub-groups.

### Bivariate analysis

Table 2 shows the average survival duration and the bivariate association of old-age mortality with the individual-, family- and community-level determinants. Most of the individual and household level factors in 2005 were associated with old-age mortality between 2005 and 2012. The community’s education level, poverty status, and social standard were significantly associated with old-age mortality. Moreover, the mortality hazard was also significantly associated with the type and geographical region of the community.

**Table 2:** Bivariate association of individual-level, family-level and community-level characteristics with mortality among older adults in India between 2005 and 2012.

Characteristics	Survival duration (in years)		Log-rank test			
	Mean	95% CI	OD	ED	Statistic	p-value
***Individual-level characteristics***						
**Age-group (in years)**						
60–69	7.41	(7.38, 7.44)	1948	3001	1568.09	< 0.001
70–79	6.82	(6.77, 6.87)	1740	1299		
80 and above	5.94	(5.83, 6.04)	997	385		
**Gender**						
Female	7.16	(7.13, 7.20)	2176	2376	35.86	< 0.001
Male	7.02	(6.98, 7.05)	2509	2309		
**Cardiovascular diseases**						
No	7.10	(7.07, 7.12)	4563	4594	11.00	0.001
Yes	6.80	(6.59, 7.00)	122	91		
**Hypertension**						
No	7.09	(7.06, 7.11)	4396	4379	1.09	0.297
Yes	7.13	(7.03, 7.23)	289	306		
**Diabetes**						
No	7.09	(7.07, 7.12)	4496	4518	3.06	0.080
Yes	6.97	(6.82, 7.11)	189	167		
**Respiratory illnesses**						
No	7.11	(7.08, 7.13)	4434	4526	58.15	< 0.001
Yes	6.62	(6.46, 6.78)	251	159		
**Other chronic illnesses**						
No	7.12	(7.09, 7.14)	4322	4432	53.04	< 0.001
Yes	6.63	(6.50, 6.77)	363	253		
**Activities of daily living**						
No disability	7.14	(7.11, 7.17)	3979	4191	107.31	< 0.001
Has disability	6.70	(6.61, 6.79)	706	494		
**Smokes tobacco**						
No	7.10	(7.07, 7.12)	3768	3822	4.34	0.037
Yes	7.06	(7.00, 7.12)	917	863		
**Drinks alcohol**						
No	7.08	(7.06, 7.11)	4378	4347	3.31	0.069
Yes	7.18	(7.08, 7.27)	307	338		
**Marital status**						
Currently married	7.24	(7.20, 7.27)	2513	3008	252.63	< 0.001
Widowed	6.83	(6.78, 6.87)	2119	1614		
Currently not married	7.36	(7.18, 7.54)	53	63		
**Level of education**						
More than 10 years of schooling	7.26	(7.17, 7.35)	292	361	67.83	< 0.001
6–10 years of schooling	7.25	(7.19, 7.32)	574	718		
Less than 5 years of schooling	7.12	(7.06, 7.18)	785	816		
No formal schooling	7.02	(6.98, 7.05)	3034	2790		
**Working status**						
Working	7.37	(7.34, 7.41)	1358	1957	330.76	< 0.001
Not working	6.90	(6.86, 6.94)	3327	2728		
**Participates in social groups**						
Yes	7.11	(7.07, 7.16)	1592	1658	4.23	0.040
No	7.08	(7.04, 7.11)	3093	3027		
**Headship status**						
Household head	7.17	(7.13, 7.20)	2081	2266	30.59	< 0.001
Not household head	7.02	(6.98, 7.06)	2604	2419		
***Family-level characteristics***						
**Family structure**						
Single generation	7.33	(7.26, 7.40)	382	526	113.61	< 0.001
Nuclear	7.33	(7.26, 7.40)	382	536		
Joint/extended	7.02	(6.99, 7.05)	3921	3623		
**Number of children in household**						
Three and more	7.07	(7.02, 7.12)	1278	1254	11.93	0.008
Two	7.04	(6.98, 7.10)	999	936		
One	7.09	(7.02, 7.15)	838	823		
None	7.13	(7.09, 7.17)	1570	1673		
**Household wealth quintile**						
Richest	7.20	(7.15, 7.25)	1114	1286	38.96	< 0.001
Rich	7.07	(7.01, 7.12)	1028	1003		
Middle	7.10	(7.04, 7.16)	933	920		
Poor	7.03	(6.97, 7.10)	823	771		
Poorest	6.98	(6.91, 7.05)	787	704		
**Household poverty**						
Not below poverty line	7.12	(7.09, 7.15)	3596	3750	33.15	< 0.001
Below poverty line	6.96	(6.90, 7.02)	1089	935		
**Caste of household head**						
Others	7.14	(7.10, 7.19)	1597	1694	33.70	< 0.001
Other backward classes	7.13	(7.09, 7.17)	1823	1889		
Scheduled castes	6.96	(6.90, 7.03)	936	820		
Scheduled tribes	6.86	(6.75, 6.98)	329	282		
**Religion of household head**						
Hinduism	7.09	(7.06, 7.12)	3837	3838	0.34	0.843
Islam	7.08	(7.00, 7.17)	464	455		
Others	7.09	(7.00, 7.18)	384	392		
***Community-level characteristics***						
**Education level of community**						
Low	7.05	(7.00, 7.09)	1633	1511	16.96	< 0.001
Medium	7.11	(7.06, 7.15)	1729	1759		
High	7.11	(7.07, 7.16)	1323	1414		
**Poverty status of community**						
Low	7.16	(7.12, 7.21)	1374	1504	17.89	< 0.001
Medium	7.07	(7.02, 7.11)	1690	1645		
High	7.04	(6.99, 7.09)	1621	1536		
**Social standard of community**						
High	7.14	(7.10, 7.19)	1460	1578	17.19	< 0.001
Medium	7.09	(7.05, 7.14)	1636	1623		
Low	7.03	(6.99, 7.08)	1589	1484		
**Type of community**						
Urban	7.15	(7.10, 7.20)	1248	1350	11.32	0.001
Rural	7.07	(7.03, 7.10)	3437	3335		
**Geographical region**						
Southern	7.13	(7.08, 7.18)	1045	1123	34.58	< 0.001
Western	7.13	(7.06, 7.20)	658	694		
Eastern	7.07	(7.00, 7.13)	714	696		
Central	6.92	(6.82, 7.01)	540	463		
North Eastern	7.31	(7.18, 7.44)	107	146		
Northern	7.09	(7.04, 7.13)	1621	1564		
Overall	7.09	(7.06, 7.12)	4685	4685		

CI, confidence interval; OD, observed number of deaths; ED, expected number of deaths.

### Model specification

**Figure 2:**
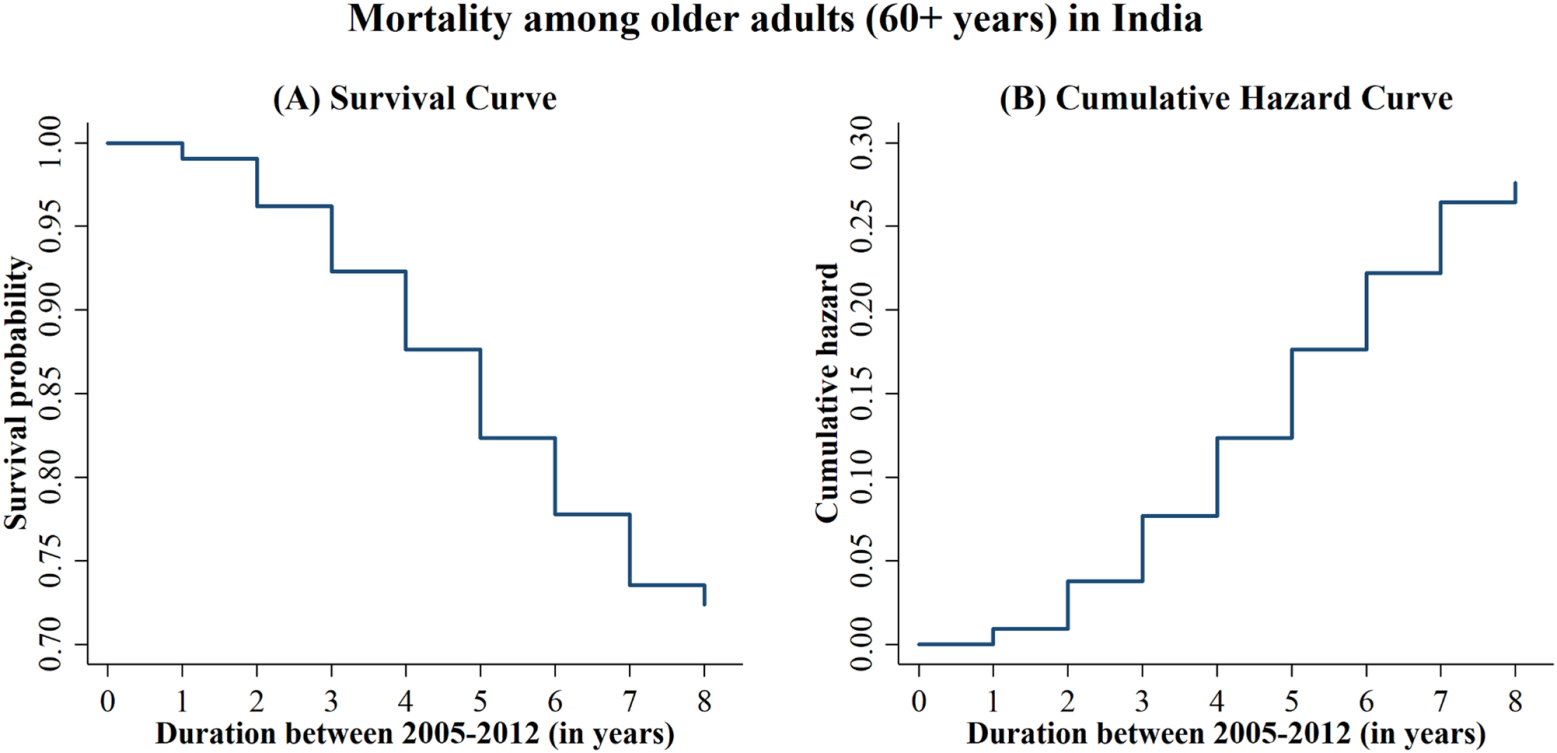
Survival plot and Cumulative Hazard plot of mortality among older adults in India between 2005 and 2012.

Table 3 shows the goodness-of-fit statistics for the Exponential, Weibull, Lognormal, Loglogistic and Gamma random-intercept survival regression models for old-age mortality. The Weibull regression models are the best fit as they have the lowest AIC and BIC scores among all the models. Further, Fig. 2 shows that the hazard of old-age mortality increases with the duration of observation. Therefore, the choice of the Weibull model is conceptually and statistically justified.

**Table 3:** Measures of goodness-of-fit for three-level random intercept survival regression models of mortality among older adults in India.

Model type	Sample (N)	Log-likelihood	AIC	BIC
**Mortality among older adults between 2005 and 2012**				
Exponential	16,964	− 19,889	39,783	39,798
Weibull	16,964	− 12,417	24,842	24,873
Lognormal	16,964	− 19,099	38,206	38,237
Loglogistic	16,964	− 19,168	38,345	38,376
Gamma	16,964	− 19,172	38,352	38,382

AIC, Akaike information criterion; BIC, Bayesian information criterion.

### Extent of old-age mortality clustering among families and communities

Table 4 shows the family- and community-level effects from the random-intercept Weibull hazard models of old-age mortality, respectively. We calculated two regression models—the null model is an empty model without any covariates, and the full model includes all covariates (see “Statistical methods”). In both models, the variation in mortality risk at both family- and community-level was statistically significant. However, the family-level variation was at least twenty times higher than the community-level variation in both models. The family-level ICC for the full model shows a 61% correlation in the risk of mortality among older adults belonging to the same family of the same community (after adjusting for the individual-level, family-level and community-level characteristics). Moreover, the median hazard of mortality is 2.12 times higher (family-level MHR) between all pairs of high-risk and low-risk families. Additionally, the statistically significant Weibull regression parameter implies that the assumption of monotonically increasing mortality hazard with time is not violated.

**Table 4:** Random-effect parameters from three-level Weibull random intercept survival regression models of mortality among older adults in India.

Measures	Null model	Full model
***Mortality among older adults between 2005 and 2012***		
**Level 3: community**		
Variance	0.03	0.03
Variance 95% CI	(0.01, 0.09)	(0.01, 0.11)
Intraclass correlation coefficient (ICC %)	3.63	2.77
Median hazard ratio (MHR)	1.19	1.18
**Level 2: family**		
Variance	0.35	0.62
Variance 95% CI	(0.24, 0.52)	(0.48, 0.80)
Intraclass correlation coefficient (ICC %)	43.06	60.75
Median hazard ratio (MHR)	1.76	2.12
**Level 1: older adult**		
Variance	0.51	0.42
Weibull regression shape parameter (γ)	1.80	1.98
Weibull regression parameter 95% CI	(1.75, 1.86)	(1.92, 2.05)
**Likelihood ratio test statistic**	40.25	101.33
**Likelihood ratio test p-value**	< 0.001	< 0.001
**Akaike information criterion (AIC)**	24,842.45	22,829.28
**Bayesian information criterion (BIC)**	24,873.40	23,216.22
**Number of communities**	2352	2352
**Number of families**	12,891	12,891
**Number of older adults**	16,964	16,964

(a) CI, confidence interval; (b) Null model, Model without any explanatory covariates; Full model, Model with all explanatory covariates; (c) Likelihood ratio tests were performed against single-level Weibull survival regression models with the same covariates respectively.

### Multivariable association and Sensitivity analysis

Table 5 (Columns 2 and 3) gives hazard ratios of association of old-age mortality risk with the explanatory variables after adjusting for the effect of other variables and accounting for the community- and family-level unobserved heterogeneity. We found that male older adults have 1.91 times [95% CI: (1.74, 2.11)] higher chance of mortality between 2005 and 2012 than their female counterparts. Older adults with cardiovascular diseases [HR: 1.37, CI: (1.11, 1.70)], diabetes [HR: 1.48, CI: (1.24, 1.77)], respiratory illnesses [HR: 1.60, CI: (1.38, 1.87)] and any other chronic illnesses [HR: 1.64, CI: (1.38, 1.87)] were more likely to die compared to those without the morbidity. Moreover, older adults who faced difficulty in activities of daily living and were not working had 1.26 [CI: (1.14, 1.39] and 1.63 [CI: (1.50, 1.76)] times greater hazard of experiencing mortality than those who had no disability and were working. The mortality hazard also increased with a decrease in education level among older adults. Interestingly, older adults who were not household heads faced an elevated risk of mortality [HR: 1.24, CI: (1.13, 1.36)] versus those who had headship.

**Table 5:** Multivariate association between risk factors with mortality among older adults in India and sensitivity analysis of the determinants.

Characteristics	Mortality among older adults between 2005 and 2012			
	Multilevel regression		Sensitivity analysis	
	HR	95% CI	E-value	CI limit
***Individual-level characteristics***				
**Age-group (in years)**				
60–69	(ref)			
70–79	1.96*	(1.82, 2.12)	2.56	2.38
80 and above	3.81*	(3.43, 4.24)	4.40	4.05
**Gender**				
Female	(ref)			
Male	1.91*	(1.74, 2.11)	2.51	2.29
**Cardiovascular diseases**				
No	(ref)			
Yes	1.37*	(1.11, 1.70)	1.79	1.34
**Hypertension**				
No	(ref)			
Yes	0.91	(0.78, 1.05)	1.34	1.00
**Diabetes**				
No	(ref)			
Yes	1.48*	(1.24, 1.77)	1.95	1.58
**Respiratory illnesses**				
No	(ref)			
Yes	1.60*	(1.38, 1.87)	2.12	1.79
**Other chronic illnesses**				
No	(ref)			
Yes	1.64*	(1.44, 1.86)	2.16	1.89
**Activities of daily living**				
No disability	(ref)			
Has disability	1.26*	(1.14, 1.39)	1.63	1.42
**Smokes tobacco**				
No	(ref)			
Yes	1.16*	(1.05, 1.28)	1.45	1.22
**Drinks alcohol**				
No	(ref)			
Yes	0.91	(0.79, 1.04)	1.35	1.00
**Marital status**				
Currently married	(ref)			
Widowed	1.38*	(1.27, 1.50)	1.81	1.64
Currently not married	0.84	(0.62, 1.15)	1.50	1.00
**Level of education**				
More than 10 years of schooling	(ref)			
6–10 years of schooling	1.12	(0.95, 1.31)	1.37	1.00
Less than 5 years of schooling	1.18*	(1.01, 1.38)	1.50	1.08
No formal schooling	1.32*	(1.14, 1.53)	1.72	1.41
**Working status**				
Working	(ref)			
Not working	1.63*	(1.50, 1.76)	2.15	1.97
**Participates in social groups**				
Yes	(ref)			
No	1.02	(0.95, 1.11)	1.15	1.00
**Headship status**				
Household head	(ref)			
Not household head	1.24*	(1.13, 1.36)	1.59	1.40
***Family-level characteristics***				
**Family structure**				
Single generation	(ref)			
Nuclear	1.28*	(1.09, 1.51)	1.66	1.30
Joint/Extended	1.50*	(1.29, 1.74)	1.98	1.67
**Number of children in household**				
Three and more	(ref)			
Two	1.07	(0.97, 1.19)	1.28	1.00
One	1.11	(1.00, 1.23)	1.35	1.00
None	1.20*	(1.08, 1.34)	1.53	1.29
**Household wealth quintile**				
Richest	(ref)			
Rich	1.21*	(1.08, 1.34)	1.54	1.30
Middle	1.15*	(1.02, 1.30)	1.44	1.13
Poor	1.25*	(1.09, 1.43)	1.61	1.32
Poorest	1.30*	(1.12, 1.51)	1.69	1.37
**Household poverty**				
Not below poverty line	(ref)			
Below poverty line	1.13*	(1.03, 1.25)	1.40	1.15
**Caste of household head**				
Others	(ref)			
Other Backward Classes	1.04	(0.95, 1.13)	1.19	1.00
Scheduled Castes	1.21*	(1.08, 1.35)	1.54	1.29
Scheduled Tribes	1.37*	(1.16, 1.62)	1.79	1.44
**Religion of household head**				
Hinduism	(ref)			
Islam	1.09	(0.97, 1.23)	1.32	1.00
Others	1.02	(0.89, 1.16)	1.12	1.00
***Community-level characteristics***				
**Education level of community**				
Low	(ref)			
Medium	0.95	(0.87, 1.04)	1.23	1.00
High	1.03	(0.92, 1.15)	1.16	1.00
**Poverty status of community**				
Low	(ref)			
Medium	1.08	(0.99, 1.19)	1.31	1.00
High	1.04	(0.94, 1.16)	1.20	1.00
**Social standard of community**				
High	(ref)			
Medium	1.07	(0.98, 1.17)	1.28	1.00
Low	1.04	(0.93, 1.15)	1.18	1.00
**Type of community**				
Urban	(ref)			
Rural	1.06	(0.96, 1.17)	1.24	1.00
**Geographical region**				
Southern	(ref)			
Western	1.11	(0.99, 1.26)	1.37	1.00
Eastern	1.15*	(1.02, 1.30)	1.44	1.13
Central	1.29*	(1.12, 1.48)	1.67	1.38
North Eastern	0.77*	(0.61, 0.98)	1.68	1.09
Northern	1.19*	(1.07, 1.32)	1.50	1.26
**Number of communities**	**2352**			
**Number of families**	**12,891**			
**Number of older adults**	**16,964**			

(a) HR, hazard ratio; 95% CI, 95% confidence interval; (ref), reference category; (b) Statistical significance is denoted by asterisk where *denotes p-value < 0.05; (c) CI limit denotes 95% confidence interval limit nearest to the null value of 1.00; (d) CI limit of E-values, whose 95% CI includes the null value, is 1.00 and is not statistically significant.

Coming to family-level characteristics, we observed that older adults residing in nuclear [HR: 1.28, CI: (1.09, 1.51)] and joint/extended [HR: 1.50, CI: (1.29, 1.74)] families faced higher mortality risk than those residing in single generation households. The results also show an economic gradient in the mortality hazard among older adults. The risk of old-age mortality was 1.25 [CI: (1.09, 1.43)] and 1.30 [CI: (1.12, 1.51)] times more among individuals residing in poor and poorest wealth quintile households compared to the richest wealth quintile counterparts. Similarly, older adults from BPL households faced an elevated risk of old-age mortality at 1.13 times [CI: (1.03, 1.25)].

Contrary to the bivariate analysis, we find that the educational level, poverty status and social standard of community was not associated with mortality risk among older adults after adjusting for the effect of other independent variables and the community-level and family-level effects. However, older adults residing in communities from Northern [HR: 1.19, CI: (1.07, 1.32)] and Eastern [HR: 1.29, CI: (1.12, 1.48)] regions of India had higher mortality hazards compared to individuals residing in the Southern region.

Table 5 (columns 4 and 5) shows the sensitivity of the association between the occurrence of old-age mortality and its determinants to unobserved variable bias. Among the individual-level characteristics, we observed that the relationship of old-age mortality with—gender, cardiovascular diseases, diabetes, respiratory illnesses and other chronic morbidities, activities of daily living, smoking tobacco, work status and household headship status did not suffer from unobserved variable bias. Upon observing family-level characteristics, it was evident that the association of family, household wealth quintile and poverty status with old-age mortality was not sensitive to omitted variable bias.

**Figure 3:**
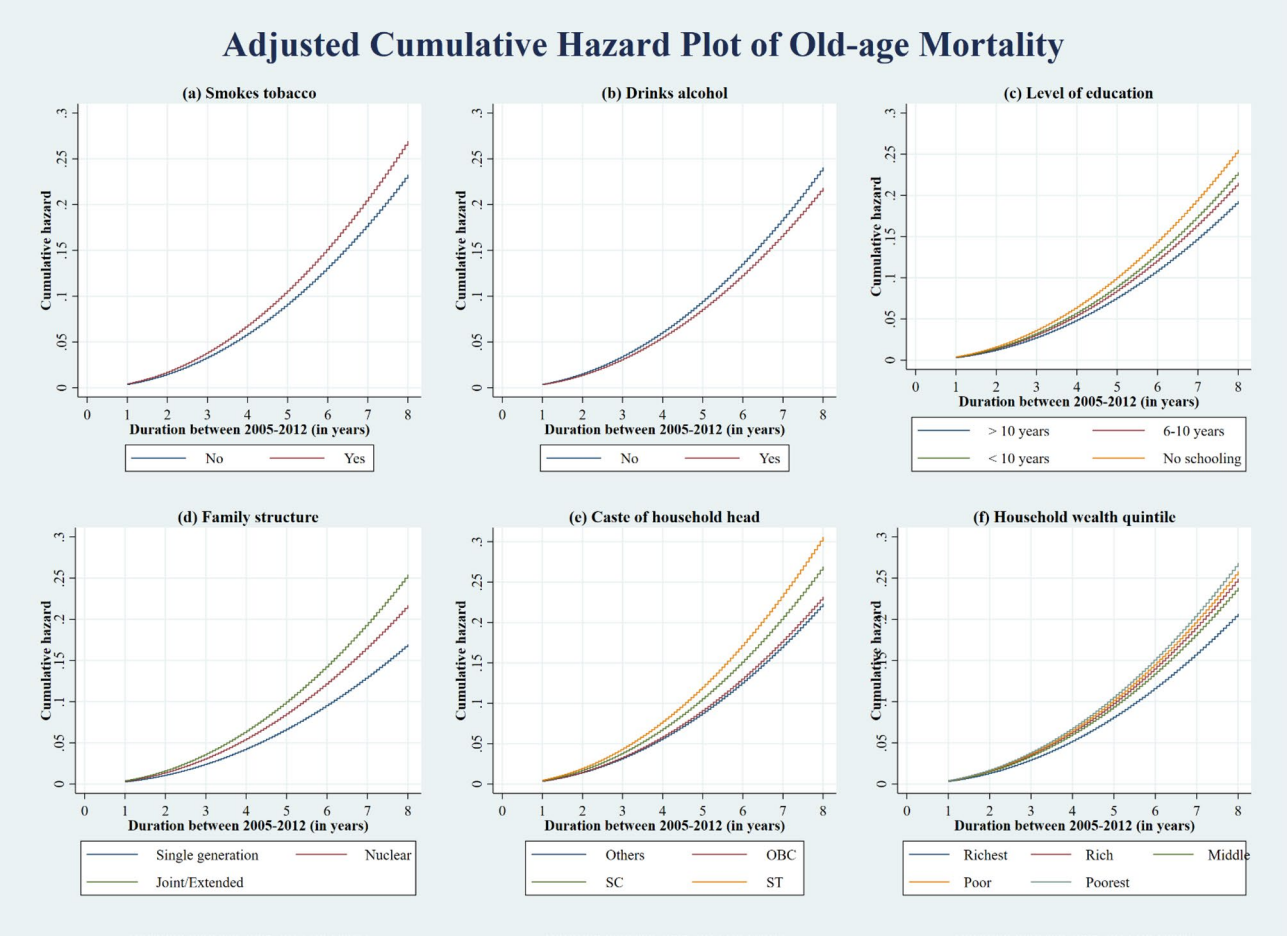
Adjusted Cumulative Hazard plot of old-age mortality by lifestyle, social and economic characteristics in India between 2005 and 2012.

After estimating the Weibull survival regression models, we obtained the adjusted cumulative hazard curve of old-age mortality grouped by lifestyle, social and economic characteristics (Fig. 3). Notably, the cumulative hazard curve in terms of smoking tobacco, drinking alcohol, level of education, family structure, the caste of household head and household wealth quintile were adjusted for the effect of other independent variables and the family- and community-level effects. We find that the graphs' results were in a similar direction to those obtained from the multivariable regression model.

## Discussion

Today, with substantial health advancements worldwide, people can expect to live into their sixties and beyond. Longer life has provided opportunities for older people (such as pursuing their passion, education, new career) and opened their chance of contributing towards families and communities. However, the growing mortality and health risks during old age hinder such opportunities and contributions^40^. The present study reveals a significant loss in the old-age population between 2005 and 2012 with an unequal distribution of mortality risks across families and communities.

Using a follow-up survey from India, the present study shows that many high-risk families (mortality clustering in families) in India lose multiple members in 60 years and above ages. Although older adults share common characteristics among communities, the present study does not find any significant clustering of mortality at the community level. Even after adjusting the unobserved heterogeneity at family and community levels, mortality risk was higher among older male adults than female counterparts. Consistent with the previous literature, older adults with poor education and those residing in unemployed condition experiences higher mortality risk^13,41^. Long term consequence of widowhood status was prominent in the study as being widow brings higher mortality risks among older adults. Such misfortunate widowhood condition is also visible from extant Indian literature^42^. The possible explanation for such association includes the protective effect of marriages in social, psychological, economic and environmental support^43^. Household headship provides constant involvement and control on the household’s social, financial affairs and a sense of security and authority. This might be the reason that household headship in older adults prevents long term mortality risk in the present study.

Ample evidence reveals an essential role of the social participation of older adults on long term survival as it may protect them from loneliness, depression, stress, or sadness of being away from loved ones^44^. However, in contrast to past evidence, the present study found an insignificant association between social participation in the first wave and mortality risk until the follow-up period. Such association might be possible due to the long-term window of observation. Since the older adults actively indulged in social activities might not continue due to poor health, leaving them in distress which can turn to a shorter lifespan. The health status of older adults is the prominent predictor among all the individual factors^10,45^. For instance, if an individual had poor health status in the first wave (i.e., chronic diseases or difficulty doing daily activities) then, better education, working status, marital status, or social participation will not be much helpful in reducing the long-term mortality risk until and unless they take early preventive measures.

Extended or joint families experience higher mortality risks among older adults. Such association is possible as the joint or extended families will have more older adults than a single or nuclear generation, making them vulnerable to mortality risks. Moreover, having no children can also be responsible for higher old-age mortality risks due to financial insecurity and loneliness^46^. Past evidence from India shows a socioeconomic disparity in older-age mortality which is also evident in this study; however, they were unable to show long term impact^20^. Consistent with a longitudinal study from Taiwan, the present study found that the most prosperous older adults and those living above the poverty line in the first wave enjoy a longer lifespan in the future^10^. Despite a higher proportion of older adults in southern regions of India, mortality risks were higher in central, northern and eastern areas of India. This might be possible due to individuals’ better health care-seeking behavior in southern regions of India^47^. However, despite having a poor health care system in the north-eastern regions, the mortality risk remains lower in the present study. Surprisingly, this may be due to the family level factors acting as a protective shield for the older adults or a higher female population in older ages^48,49^. For instance, the solid biological advantage of females and satisfaction of being closer to family and community might help in lowering the mortality risk of north-eastern older adults.

Despite providing robust evidence of heterogeneity in older-age mortality risk at the family level and revealing the long-term effect of individual, household and community factors on older-age mortality risks, the present study has its limitations. Ample evidence shows that depression and life satisfaction are emerging as prominent indicators of a longer lifespan; however, we could not capture their effect due to the unavailability of information in the data used for this study^50^. Self-reported chronic conditions may create multiple problems in the form of biases like the accuracy of responses, so biological or clinical markers of chronic diseases should be considered while understanding the mortality dynamics of the individual^51^. The present study uses self-reported information of chronic diseases as the biological measures of older adults were unavailable. Additionally, the study results are unweighted and need to be interpreted accordingly.

## Conclusion

In India, families are the prime source of caregivers for older adults. With significantly higher mortality risk heterogeneity across Indian families, the present study confirms that the familial-level factors (i.e., having children, income-level, poverty status and ethnicity) in early years of life may have a noticeable impact on the longer lifespan of older adults. Along with the individual-level factors (i.e., education, employment, support of a partner, social participation, and health behavior), health status in the form of chronic diseases and daily living activities remains to have a significant impact on the survival of older adults.

Past literature from developed countries shows no health gradient among rich and poor before the enlightenment of science and advanced technologies^2^. However, with the growing development of treatments and drugs, a wealthier population pays quickly to cure diseases and ensure a longer life. This trend continues in developing countries, too, combined with the lesser knowledge of health behavior among the uneducated population, increasing the disparities across socioeconomic statuses. Such past evidence and the detrimental effect of poverty and lower income in the present study confirms the unequal share of mortality distribution in old-age across families. The present study will help the policymakers understand the development of such a mortality gradient in the old-age population of India and provide efficient evidence of policy interventions across high-risk families. The long-term consequences of socioeconomic status and health conditions on old-age mortality risk further urge early life interventions as those started in late-life might have negligible impact on keeping the older adults alive and healthy.

Traditionally, joint or extended families were one of the characteristics of Indian life where older adults enjoy authority along with care from younger generations. However, changes in living arrangements and lifestyle in past years bring a shift towards the caregiver role in families. The emergence of new health conditions like life satisfaction, stress, and depression among wealthy and low-income families, further, urges future research on the old-age mortality risks in India.

## Data Availability

The datasets used for this study are publicly available from the Inter-university Consortium for Political and Social Research (ICPSR) data repository^52,53^.
